# Airway Management of the Patient with Maxillofacial Trauma: Review of the Literature and Suggested Clinical Approach

**DOI:** 10.1155/2015/724032

**Published:** 2015-06-16

**Authors:** Michal Barak, Hany Bahouth, Yoav Leiser, Imad Abu El-Naaj

**Affiliations:** ^1^Department of Anesthesiology, Rambam Health Care Campus, and the Ruth and Bruce Rappaport Faculty of Medicine, Technion-Israel Institute of Technology, 31069 Haifa, Israel; ^2^Trauma Center & Emergency Surgery, Department of General Surgery, Rambam Health Care Campus, 31096 Haifa, Israel; ^3^Department of Oral and Maxillofacial Surgery, Rambam Health Care Campus, 31096 Haifa, Israel; ^4^Department of Oral and Maxillofacial Surgery, Baruch Padeh Medical Center, Affiliated to the Faculty of Medicine of Bar-Ilan University, Poriya, 15208 Tiberias, Israel

## Abstract

According to the Advanced Trauma Life Support recommendations for managing patients with life-threatening injuries, securing the airway is the first task of a primary caregiver. Airway management of patients with maxillofacial trauma is complex and crucial because it can dictate a patient's survival. Securing the airway of patients with maxillofacial trauma is often extremely difficult because the trauma involves the patient's airway and their breathing is compromised. In these patients, mask ventilation and endotracheal intubation are anticipated to be difficult. Additionally, some of these patients may not yet have been cleared of a cervical spine injury, and all are regarded as having a full stomach and having an increased risk of regurgitation and pulmonary aspiration. The requirements of the intended maxillofacial operation may often preclude the use of an oral intubation tube, and alternative methods for securing the airway should be considered before the start of the surgery. In order to improve the clinical outcome of patients with maxillofacial trauma, cooperation between maxillofacial surgeons, anesthesiologists, and trauma specialists is needed. In this review, we discuss the complexity and difficulties of securing the airway of patients with maxillofacial trauma and present our approach for airway management of such patients.

## 1. Introduction

The patient with maxillofacial trauma presents serious challenges for the physician because airway management in these patients can be complicated by their injury. The first challenge is to secure the airway for sufficient and effective breathing and/or ventilation. When planning to secure the airway, the physician has to consider several aspects: (a) the nature of the trauma and its effect on the airways, (b) potential difficulties in mask ventilation or endotracheal intubation, (c) possible trauma of the cervical spine, (d) the risk of regurgitation and aspiration of gastric contents, (e) significant bleeding that precludes view of airway anatomy and may cause circulatory deterioration, and (f) the type of maxillofacial operation that is to be done and whether the oral cavity needs to be empty for performing the procedure and closed with maxilla-mandibular fixation (MMF) at the end of surgery. The time available for deciding on and then performing the optimal method in order to secure the airway under a particular set of circumstances is often short because the patient's condition can deteriorate quickly.

In this review we will describe and discuss the various stages of airway management of the patient with maxillofacial trauma and how each stage contributes to comprehensive, safe, and practical airway management of these patients.

## 2. Maxillofacial Trauma and Airway Injuries

Safe and optimal airway management of the patient with maxillofacial trauma requires appreciation of the nature of the trauma. There are several maxillofacial injuries that require immediate treatment, especially in acute upper airway compromise and/or when profuse hemorrhage occurs. According to Hutchison et al. [1], there are six specific situations associated with maxillofacial trauma, which can adversely affect the airway.

1. Posteroinferior displacement of a fractured maxilla parallel to the inclined plane of the base of the skull may block the nasopharyngeal airway.
2. A bilateral fracture of the anterior mandible may cause the fractured symphysis and the tongue to slide posteriorly and block the oropharynx in the supine patient.
3. Fractured or exfoliated teeth, bone fragments, vomitus, blood, and secretions as well as foreign bodies, such as dentures, debris, and shrapnel, may block the airway anywhere along the oropharynx and larynx.
4. Hemorrhage from distinct vessels in open wounds or severe nasal bleeding from complex blood supply of the nose may also contribute to airway obstruction.
5. Soft tissue swelling and edema which result from trauma of the head and neck may cause delayed airway compromise.
6. Trauma of the larynx and trachea may cause swelling and displacement of structures, such as the epiglottis, arytenoid cartilages, and vocal cords, thereby increasing the risk of cervical airway obstruction.

A high index of suspicion, a meticulous physical examination, and close observation of the patient may assist in the early detection of such situations and facilitate proper and timely management in order to avoid future complications. Once airway management has been completed and hemorrhage is controlled at all sites, the patient should have a computerized tomography (CT) scan of the head and neck with i.v. contrast material, in order to demonstrate the vascular structures surrounding the injury sites and provide detailed information on the type and extent of the trauma, for definitive management of bone and soft tissue injuries. The imaging and the definitive maxillofacial operation may be deferred until all life- and/or organ-threatening injuries have been properly managed.

## 3. Early Airway Maintenance

According to the Advanced Trauma Life Support (ATLS) recommendations for managing patients who sustained life-threatening injuries, airway maintenance with cervical spine immobilization is the first priority [2]. The loss of an airway may be lethal and can occur faster than the loss of the ability to breathe or the onset of circulatory problems. Thus, lifesaving intervention should begin with airway management, when required [2–4]. In fact, the most common critical care errors that contribute to the death of trauma patients are related to airway and respiratory management [5]. Airway management problems are not confined to the early stages of the “triage process” or to the resuscitation of the patient. Morbidity and mortality of in-hospital trauma patients often result from critical care errors, with airway management being the most common [5, 6]. Gruen et al. studied the causes of death of 2594 trauma patients in order to identify the error patterns which contributed to inpatient deaths [6]. They found that 16% of inpatient deaths were caused by failure to intubate or failure to secure or protect the airway.

The first action in the process of early airway management is preoxygenation, which may prolong the time interval up to hypoxemic state. Effective preoxygenation of the lungs increases oxygen content in the functional residual capacity which is the principal oxygen store during apnea. Since the time for achieving airway control before onset of dangerous levels of hypoxemia is critical, preoxygenation is crucial and is to be carried out as much as possible, using a nonrebreathing mask. In some patients preoxygenation is unfeasible due to the maxillofacial trauma itself, and hypoxemia is to be expected.

Endotracheal intubation is the gold standard procedure to secure the airway in trauma patients. It is to be performed via the oral route with a rapid sequence induction and a manual in-line stabilization maneuver, in order to decrease the risk of pulmonary aspiration and take into account a potential cervical spine (C-spine) injury [2]. However, endotracheal intubation is expected to be difficult in a maxillofacial trauma patient. The challenge in performing the intubation arises mainly from a difficulty in viewing the vocal cords using conventional direct laryngoscope. The oral cavity, pharynx, and larynx may be filled with blood, secretions, soft tissue, and bone fragments, all of which preclude a good view of the vocal cords.

Regarding mask ventilation, mask ventilation is problematical in the patient with maxillofacial trauma because the oral cavity and/or oropharynx's anatomy could be disarranged by the trauma and/or blocked by bleeding. Thus, the ventilation mask cannot be properly fitted to the face for effective mask ventilation. Furthermore, an injured airway may prevent efficient air transfer from the mask to the lungs.

In addition to the problem of anticipated difficult intubation and difficult mask ventilation, several other factors may aggravate the scenario: the risk of regurgitation and aspiration, the potential C-spine injury, the patient who is starved for air and may already be hypoxemic, could also be uncontrollable and combative, and lack of experience of the primary care provider.

### 3.1. Full Stomach

Like all trauma patients, the patient with maxillofacial trauma must be assumed to have a “full stomach” because digestion stops when the trauma occurred. Since such patients often bleed from the upper airway, blood is swallowed and accumulates in the stomach. Accordingly, the risk of regurgitation and aspiration is high. In order to diminish such risks, evacuating the contents of the stomach through the nasogastric tube before proceeding with airway management is recommended. However, insertion of a nasogastric tube in a confused, uncooperative, sometimes intoxicated patient who has sustained a facial injury may, by itself, trigger vomiting. In addition, it is relatively contraindicated in cases with a possible fracture of the base of skull. Formerly it was accustomed to use Sellick's maneuver [7], in order to reduce the risk of pulmonary aspiration. The Sellick's maneuver is a technique in which the esophagus is occluded by applying pressure on the cricoid cartilage. Over the years Sellick's maneuver, which is also called cricoid pressure, has been incorporated into “rapid sequence induction” (RSI). Although Sellick's maneuver and RSI are widely used, the maneuver may significantly hamper endotracheal intubation because the laryngeal view is worsened [8, 9]. In addition, its efficacy in preventing aspiration is questionable [10], and in some cases it may lead to ruptured esophagus. Thus, the application of cricoid pressure as prophylaxes for aspiration in trauma patients is no longer indicated [11].

### 3.2. C-Spine Injury

A patient with a supraclavicular injury is considered to have a C-spine injury, until proven otherwise by imaging [12, 13]. Since a complete C-spine clearance may take several hours and sometimes days to achieve, the patient must be fitted with a neck collar for cervical spine immobilization. At the time of intubation, the anesthesiologist's assistant performs “in-line stabilization” in order to support the head and neck in place and prevent neck movement throughout the procedure [14]. However, several studies indicate that direct laryngoscopy and intubation are unlikely to cause clinically significant neck movements. On the other hand, “in-line stabilization” may not always immobilize the injured segments effectively. In addition, “in-line stabilization” worsens the laryngoscopic view which may, in turn, worsen the outcome in traumatic brain injury by delaying endotracheal intubation and causing hypoxia [15, 16]. Using a video laryngoscope, instead of a conventional laryngoscope with a Macintosh blade, may be beneficial for intubating patients whose neck position needs to be in a neutral position and their cervical spine requires immobilization [17–19]. Neck movements during laryngoscopy using a conventional Macintosh laryngoscope has been compared to that using the GlideScope video laryngoscope [18] and the Truview PCD laryngoscope [19]. The results of the two studies found that the number of neck movements is reduced when using the video laryngoscopes for endotracheal intubation.

#### 3.2.1. Maxillofacial Bleeding

In patients with major maxillofacial trauma, severe uncontrolled bleeding is possible, especially in trauma that involves more than two thirds of the face, “panfacial trauma.” Since the head and neck region is abundantly vascularized, severe life-threatening bleeding may occur during isolated facial trauma [20, 21]. The hemorrhage affects the patient's condition and prognosis in several ways: (a) blood in the oral cavity often excludes mask ventilation, (b) it may preclude good view of airway anatomy, thus making intubation very difficult, (c) significant hemorrhage may cause circulatory compromise that may be fatal, (d) coagulation may deteriorate due to massive blood transfusion, and (e) the surgical field conditions during bleeding are less than optimal for operating. Management of the patient includes volume replacement and local control of the bleeding with packing, ligation, or, in selected cases, arterial embolization [22, 23].

### 3.3. Emergency Situations

Managing the airway in an emergent situation poses additional difficulty because the time to accomplish the task is short and the patient's condition may deteriorate quickly. Both decision-taking and performance are diminished at such times. The performance of urgent or emergent intubation is associated with remarkably high complication rates, which may exceed 20% [24, 25]. These high rates are due to several factors, which include repeated intubation attempts, the need to perform direct laryngoscopy without muscle relaxation, and the lack of experience of the operator. The main complications that may occur at that time are hypoxemia, aspiration, esophageal intubation, esophageal tear, alterations in the heart rate, new onset cardiac dysrhythmias, and cardiac arrest.

### 3.4. Personnel Experience

In emergency situations, the care of acute trauma patients is provided by individuals who are often not experienced, the “inverse care law” [26]. The responsibility for acute airway management often falls into the hands of nonanesthesiologists [27, 28]. In their multicenter analysis of 8937 intubations in the emergency department, Walls et al. [28] reported that anesthesiologists performed only 3% of the intubations, and the remaining 97% of the intubations were performed by emergency physicians (87%) and physicians from other specialties (10%). In order to improve the clinical outcome of patients with maxillofacial trauma, we believe that the most experienced personnel in the hospital should be tasked with airway management of such patients.

## 4. Approach to the Airway of the Patient with Maxillofacial Trauma

### 4.1. Airway Evaluation and Preparation

Airway evaluation of a patient with maxillofacial trauma should be done thoroughly and as quickly as possible because the patient's airway is compromised. Additionally, the attending physicians should become familiar with all details of the trauma and identify the difficulties involved in order to choose the best approach for managing the patient's airway [29, 30]. Team work between the surgeons, the anesthesiologists, and the trauma specialists is necessary for managing the patient.

At this time we ask the following questions.

- Is the patient conscious? If so, the use of sedatives or analgesics should be done cautiously, if at all, because the airway can be lost following injudicious use of such drugs [31].
- Is the patient breathing spontaneously? If so, preoxygenation is mandatory. There is time to arrive at the hospital and manage the airway under the best conditions, with the best equipment and by the most experienced personnel. Failed attempts at endotracheal intubation by inexperienced or nonexpert individuals could cause rapid deterioration in the patient's condition. According to the American Society of Anesthesiologists (ASA) Practice Guidelines for management of the difficult airway, spontaneous breathing should be preserved in patients with anticipated difficult endotracheal intubation [32].
- Is the patient hypoxemic? If preoxygenation is possible and effective in improving patient's oxygenation then it is to be done with a face mask. If preoxygenation is not possible then ventilation is to be pursued at that time by the caretakers, according to their capability and equipment.
- What is the extent, the details, and the anatomy of the injury? Are the bony structures of the face involved? In cases of massive injuries, mask ventilation may be impossible, while injury limited to the soft tissues may enable mask ventilation [33].
- For quick and easy identification of factors that may predispose difficult intubation or ventilation, one may use the LEMON assessment [33, 34]. The components of this assessment are as follows: look externally to detect difficult airway predictors, such as short neck and evaluate mouth opening and thyromental distance, Mallampati class, obstruction of the upper airway that may be noticed by stridor, and neck mobility. If one or more of the components are degraded then difficulty in airway control is to be expected.
- Is there a limitation of mouth opening? If so, is pain the cause of the limitation and can the mouth be opened wider after analgesia? The answers to these questions depend, among other things, on whether there is the clinical or radiological evidence of a temporomandibular joint (TMJ) injury. If the limitation of mouth opening is caused by a TMJ injury, sedation will not improve mouth opening and may even worsen the scenario.
- Are there additional predictors for difficult endotracheal intubation, such as obesity? In their study of 1377 intubations in the emergency department patients, Gaither et al. identified C-spine immobility, blood or vomitus in the airway, airway edema, facial or neck injury, and obesity as predictors of difficult endotracheal intubation [35].
- What are the requirements of the upcoming maxillofacial surgery? Does the oral cavity need to be completely free of any medical devices for performing the surgery?

As with all situations of difficult airway management, the staff should be notified and prepared. The patient should be transferred as quickly as possible to a dedicated location, in the emergency department or the operating rooms, where the best equipment and conditions are available for performing endotracheal intubation. That location is to be equipped with all available airway management tools, including laryngoscopes of various types and sizes, video laryngoscopes, fiber-optic devices, and surgical devices for cricothyroidotomy, according to the published guidelines' difficult airway equipment list [36]. In addition, high-flow suction unit, high pressure blood heaters and transfusers, and resuscitation equipment are to be prepared and ready when there is a call.

### 4.2. Airway Management Devices

There are numerous airway management devices; however, only an endotracheal tube or tracheostomy tube is considered to be definitive when applied. As stated earlier, not having an unobstructed view of the vocal cords of the patient with maxillofacial trauma is the main obstacle for performing successful endotracheal intubation in such patients. Numerous airway devices and strategies have been developed to overcome this obstacle. Some devices, such as the flexible fiber-optic bronchoscope (FOB), enable an indirect view of the vocal cords. Other devices, such as the laryngeal mask airway (LMA) or the double lumen esophageal-tracheal Combitube, can be inserted blindly and do not require view of the vocal cords by any means. Another option for endotracheal intubation of a patient with maxillofacial injury is to place an LMA and then pass an endotracheal intubation tube through the LMA. The final option is the surgical one: to establish a direct access to the trachea by performing a cricothyroidotomy or a tracheotomy.

Since this review is a limited scope review, we chose to discuss several airway devices that are beneficial in the management of the patient with a maxillofacial trauma.

### 4.3. Airway Devices That Enable an Indirect View of the Vocal Cords

#### 4.3.1. The FOB

Although performing fiber-optic intubation under local anesthesia for achieving successful endotracheal intubation is one of the recommended methods in situations where airway management is difficult [32], the use of FOB is somewhat impractical in patients with maxillofacial trauma. Blood, vomitus, and secretions in the patient's airway may preclude vision by fiber-optic instruments, and accomplishing effective local anesthesia in the injured regions is difficult. Furthermore, the patient's cooperation is essential for such an approach, and this cooperation is not easy to obtain in the trauma patient.

#### 4.3.2. The Video Laryngoscope

The video laryngoscope, such as GlideScope video laryngoscope, enables an indirect view of the epiglottis and the vocal cords [37]. The successful use of a video laryngoscope relies on a good view of the inner airway, which is precluded in the trauma patient by blood and secretions. Accordingly, the use of a video laryngoscope is not better than that of FOB. However, the video laryngoscope may be useful in selected patients with soft tissue swelling at the base of the tongue, and in those patients in whom disruption of the normal anatomy precludes locating the epiglottis.

### 4.4. Blindly Placed Airway Management Devices

*Supraglottic airway devices (SAD),* such as the LMA and its several diverse variations, are very important devices for managing the difficult airway [32]. For airway management of the trauma patient, the SAD is placed blindly in the oropharynx and its successful placement requires minimal experience [38–40]. However, SADs do not provide a definitive airway and can be displaced when the patient with an SAD is moved and transferred. In addition, patients suffering from facial trauma often have minimal space in the mouth, which complicates the use of supraglottic airway device. This restricts the use of these devices in some cases. Thus, it is not a final airway tool for managing trauma patients, especially for trauma patient that requires maxillofacial surgery, where the oral cavity is to be empty. However, a SAD is an ideal rescue device for ventilating a patient until the definitive airway is achieved, as has been repeatedly proven in combat casualties and many other trauma victims [41–43]. When the definitive surgery is to be performed, the SAD may be replaced by an endotracheal tube [44] or, alternatively, into a tracheostomy.

*The Combitube* is another airway management device that is inserted blindly into the oropharynx. In a patient with a maxillofacial trauma, the use of the Combitube may result in additional damage to the upper airway. Furthermore, insertion of Combitube can be associated with serious injury to the upper airway and digestive tract, such as esophageal laceration and perforation, tongue edema, vocal cord injury, tracheal injury, aspiration pneumonitis, and pneumomediastinum [45].

### 4.5. The Surgical Airway

The surgical airway is considered to be the last option in airway management; however, in patient with facial trauma sometimes it is the best solution. To be prepared well, a qualified surgeon should stand on site during conventional airway management in order to be immediately in charge. Performing a cricothyroidotomy or tracheotomy under local anesthesia is a lifesaving procedure in selected patients in the “cannot intubate, cannot ventilate” situation [32, 46–48]. Surgical creation of an airway is a safe method for securing the airway when the procedure is done by an experienced surgeon. However, this approach has its drawbacks: it carries a 6% rate of complications such as hemorrhage or pneumothorax, in an elective scenario [49]. This procedure can be difficult to perform in an urgent or emergent situation [50, 51] and procedure can occasionally be fatal [52]. When a tracheotomy is carried out under local anesthesia, it is uncomfortable or even painful for the patient, who may already experiencing severe pain and anxiety. For the operator, especially the less experienced one, it may be extremely stressful [53, 54] and, as a rule, the procedure is best performed by the team's surgeon rather than the anesthesiologist.

**Figure 1:**
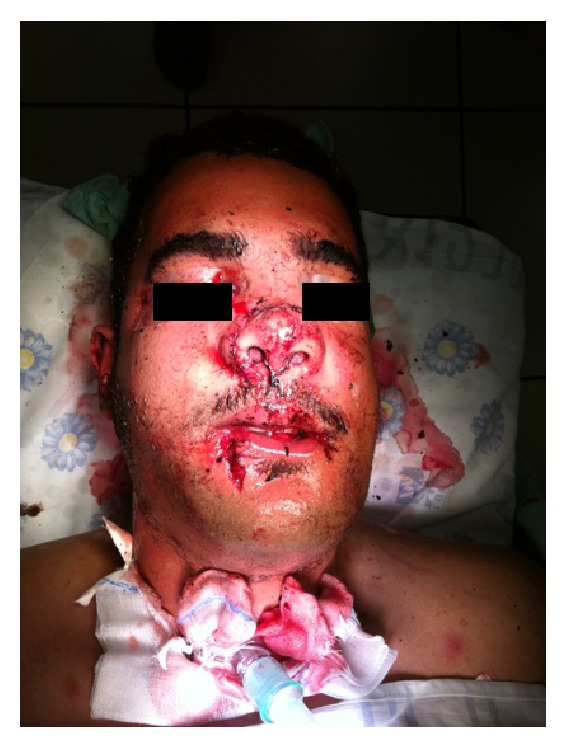
A patient with maxillofacial trauma being ventilated through tracheostomy.

Of the two surgical procedures, there seems to be a propensity for doing a tracheotomy rather than a cricothyroidotomy. In their retrospective analysis of 4312 emergent airways, Dillon et al. found that only 34 patients (0.008%) required emergency surgical access, and of these 34 patients a tracheotomy was done in 24 and a cricothyroidotomy was done in 10 patients [55]. This preference may be attributed to the higher failure risk of cricothyroidotomy [56]. Although emergency surgical access is not frequently used, the surgical airway may be the route of choice when the maxillofacial trauma is extensive and the patient requires postoperative mechanical ventilation and MMF (Figure 1).

### 4.6. The Conventional Direct Laryngoscopy

Direct laryngoscopy using a conventional laryngoscope is a simple and straightforward method for securing the airway of a patient and may be successful when done by an experienced operator. However, the risk of losing the airway is high, and hemodynamic side effects sometimes occur [57]. Considering the risk of a failed endotracheal intubation, direct laryngoscopy should be reserved for selected slim patients with good surface anatomy of the neck, where urgent cricothyroidotomy or tracheotomy is feasible when necessary, and an ear, nose, and throat specialist is ready to perform the surgical airway.

## 5. Preparing the Patient for Maxillofacial Surgery

The maxillofacial surgery is done after stabilization of the patient; the radiographic tests were performed, and all the injuries were identified. In some patients, the surgery is performed at the same time as the surgery on other injured organs. Operating on patients with a maxillofacial trauma and especially those with a severe complex comminuted panfacial fractures is quiet challenging for the surgeon. The surgeon has to perform fracture reduction, repair soft tissue injuries, and restore the occlusion. In order to facilitate optimal operating conditions and to achieve a proper pretraumatic figuration and function, the occlusion has to be maintained and checked at all times during the surgery. At the end of the surgery the mouth is to be set closed with MMF [33]. These surgical requirements preclude the use of oral endotracheal tube. In cases when MMF is not required, an oral tube may be suitable. The choice of an airway device that will be used during the operation is to be agreed upon by the surgeon who is familiar with the planned procedure, including possible intraoperative change of plan and potential postoperative complications.

**Figure 2:**
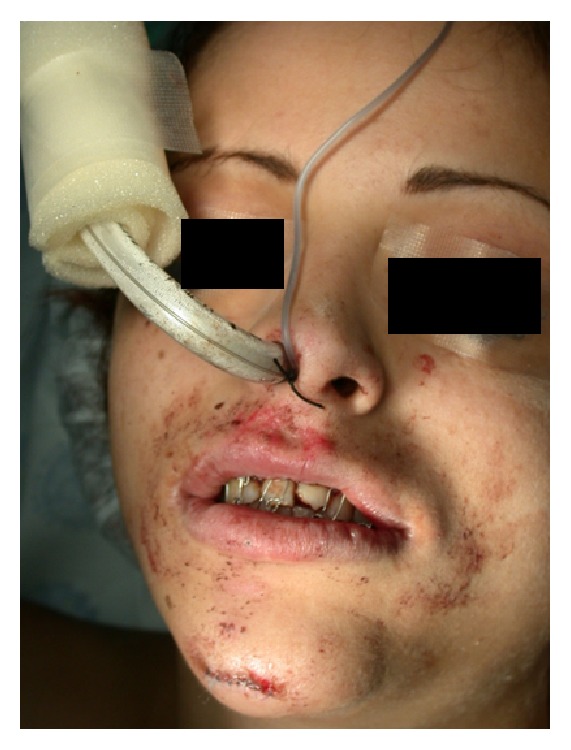
A patient with maxillofacial trauma ventilated through a nasal endotracheal tube.

At this point, a decision needs to be made on the type of airway control which is suitable for the intended surgery. Some patients arrive to the operating room conscious and spontaneously breathing and their maxillofacial trauma is not extensive. In selected patients, nasoendotracheal intubation can be used for airway control during surgery [58] (Figure 2). However, nasoendotracheal intubation is relatively contraindicated in patients with midface fractures or fractures at the base of the skull [59].

**Figure 3:**
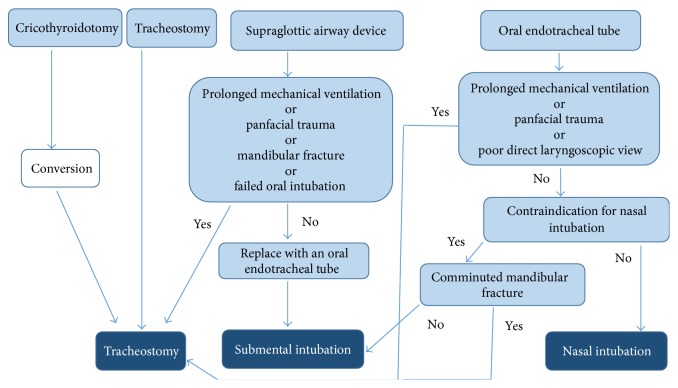
A decision-making algorithm for securing the airway of a patient with maxillofacial trauma during the maxillofacial surgery.

Severely injured major trauma patients usually arrive at the operating room with one of the following airway control devices, namely, an endotracheal tube, a SAD, a cricothyroidotomy, or a tracheotomy, that were done earlier in the field or emergency room. In order to make a decision on which method to use for airway control during the surgery, we use an algorithm which we developed and based on our experience at Rambam Health Care Campus, a level I trauma center (Figure 3). For those trauma patients where a tracheostomy or a cricothyroidotomy was performed as the first line of securing the airway it is useful subsequently for the surgery and postoperative recovery period. It is recommended, however, that cricothyroidotomy will be converted to tracheotomy at this time [60]. If the patient arrived at the operating room with an oral endotracheal tube, and prolonged ventilation is expected, the oral tube is to be changed to open tracheostomy. When the patient presents with no mandibular fracture, a contraindication to nasal intubation is presented and there is no need for prolonged intubation; submental orotracheal intubation will be used as the method for securing the airway during surgery [61–63].

### 5.1. Submental Orotracheal Intubation for Maxillofacial Surgery

Submental orotracheal intubation was developed in order to avoid the need for tracheotomy and to permit unfettered access to the oral region. This type on intubation is done (a) in patients with comminuted fracture of the midface or the nose, where nasal intubation is contraindicated, (b) in patients who require restoration of the occlusion, and (c) in patients whose condition permits extubation at the end of surgery.

However, this type of intubation is contraindicated in patients with comminuted mandibular fractures.

#### 5.1.1. Surgical Technique

**Figure 4:**
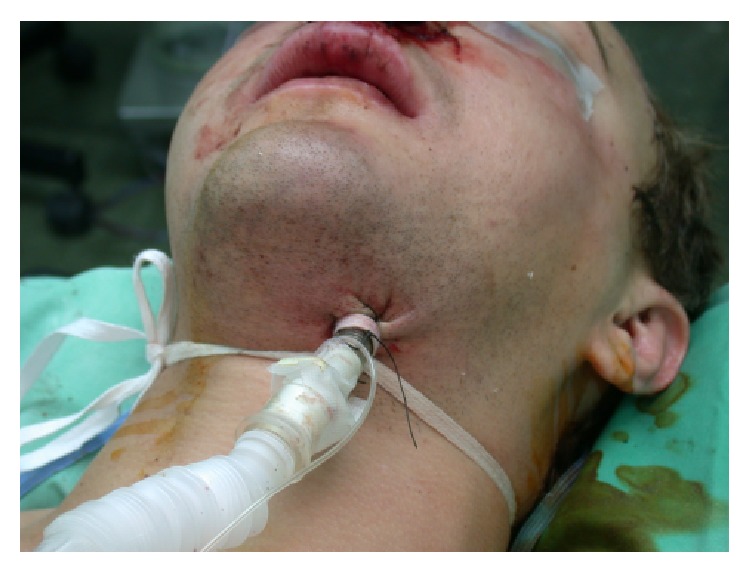
Submental orotracheal intubation in a patient with maxillofacial trauma.

Submental orotracheal intubation requires the use of a spiral reinforced armored endotracheal tube in order to prevent the tube from kinking during its usage. Following an orotracheal intubation, a 2 cm incision is made half way between the chin and the angel of the mandible, and a blunt dissection is performed to the oral floor. A surgical access is made through the superficial fascia, platysma, and deep fascia. The opening is positioned in the floor of the mouth. At the end of the dissection the forceps should be opened in order to create a tunnel for passing the tube without any interference. When creation of the surgical access is complete, the tube is pulled through the tunnel, using gentle rotational movements. Following this maneuver, the tube is connected to the ventilating machine and sutures are used to fix the tube's position (Figure 4).

When indicated, extubation is done through the external skin incision: the intermaxillary fixation is released, the fixation ligature of the tube should be opened, and the tube is disconnected from the machine. The tube should be pulled back into the oral cavity and reconnected to the anesthesia machine. The submental incision should be closed. At this point, the patient is ventilated through an oral endotracheal tube and extubation is accomplished as usual. There is no need to suture the intraoral incision and the skin incision is closed using the sutures that were placed at the time of intubation.

Complications from submental endotracheal intubation do occur and include bleeding, damage to the lingual nerve, and the marginal mandibular branch of the facial nerve, damage to the duct of the submandibular gland, damage to the sublingual gland, salivary fistulae, and skin infections [64, 65].

## 6. Postoperative Management of the Patient with Maxillofacial Trauma

The patient with a difficult airway is also at high risk for postoperative complications. Following surgery, the mucous membranes are edematous, the soft tissues are swollen, and the airway may be compressed. Neck expandability is relatively low and even a small hemorrhage in the region could result in airway compromise. The risk of airway-related complications during the perioperative period was studied by Peterson et al. [66]. They analyzed the American Society of Anesthesiologists Closed Claims database to identify the patterns of liability associated with the management of the difficult airway. They found that 12% of complications arose at extubation and 5% during recovery.

In intubated patients with maxillofacial trauma, extubation should be deferred until the edema subsides. During extubation the patient should be monitored closely and the care providers should be prepared for the possibility of reintubation. It is important to prevent nausea and vomiting because of the risk of gastric content aspiration [67], especially in those patients with MMF, because pulmonary aspiration is plausible. For those patients with a tracheotomy tube, the patient may be awakened and allowed to breathe spontaneously through the tracheostomy tube for a few days in order to ensure a safe recovery.

## 7. Conclusion

Airway management of patients with maxillofacial trauma is challenging. The clinical status and features of the trauma dictate the approach for securing the airway, and a series of steps are to be planned before airway management is initiated. Knowledge of the specific attributes of the difficult airway, expertise in the appropriate techniques for managing the difficult airway, familiarity with the various airway devices, and prompt recognition of a failed airway are necessary for optimal patient care. Skilled, open-minded personnel and a variety of advanced airway equipment are required for managing the trauma patient. Teamwork between the maxillofacial surgeon, the anesthesiologist, and the trauma expert, in which each specialist contributes his/her expert knowledge, is mandatory for better outcomes.
